# Supplementary material for: Interaction between genetic risk and comorbid conditions in endometriosis
Source: HGG Adv. 2025 May 13;6(3):100456. doi: 10.1016/j.xhgg.2025.100456 (PMC12159439; doi:10.1016/j.xhgg.2025.100456)
Supplement: Document S1. Figures S1–S6 [file mmc1.pdf]

HGGA, Volume *6*

Supplemental information

Interaction between genetic risk and comorbid conditions in endometriosis

Isabelle M. McGrath, Valentina Rukins, Triin Laisk, Estonian Biobank Research Team, Sally Mortlock, and Grant W. Montgomery

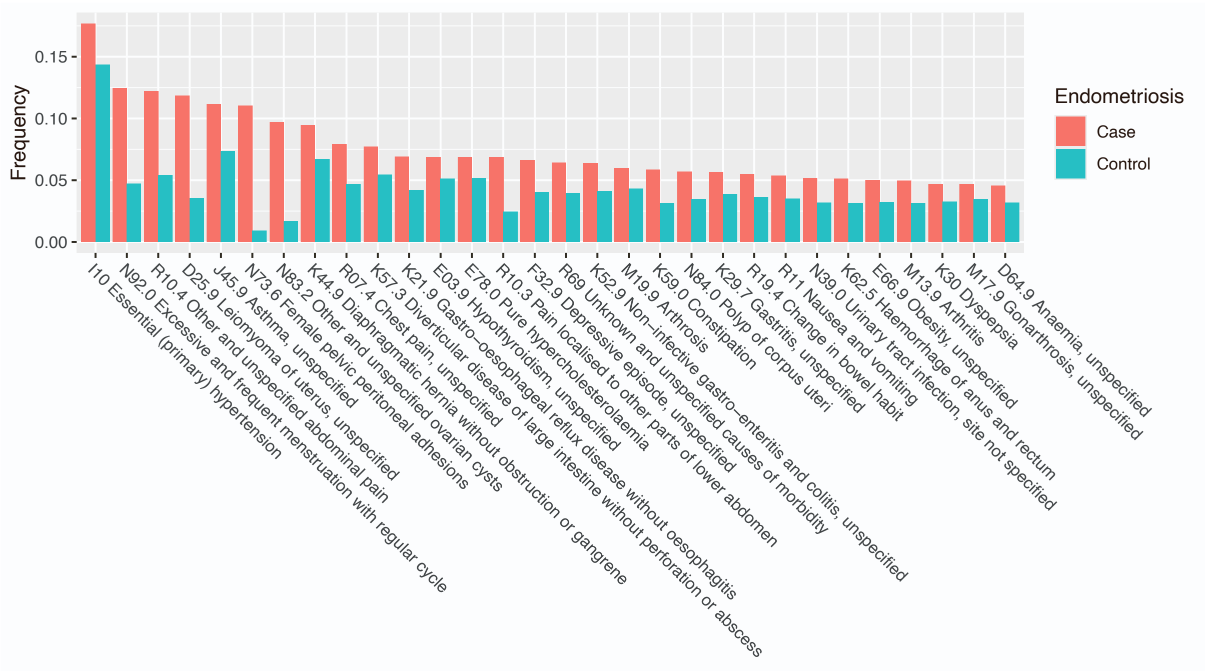

Figure S1. Prevalence of ICD10 codes significantly associated with endometriosis in 5,432 unrelated European ancestry endometriosis cases and 92,344 age matched female controls in the UK Biobank. The 30 most frequently occurring codes in endometriosis cases are shown, ordered by frequency in cases. ICD10 codes related to external factors have been excluded.

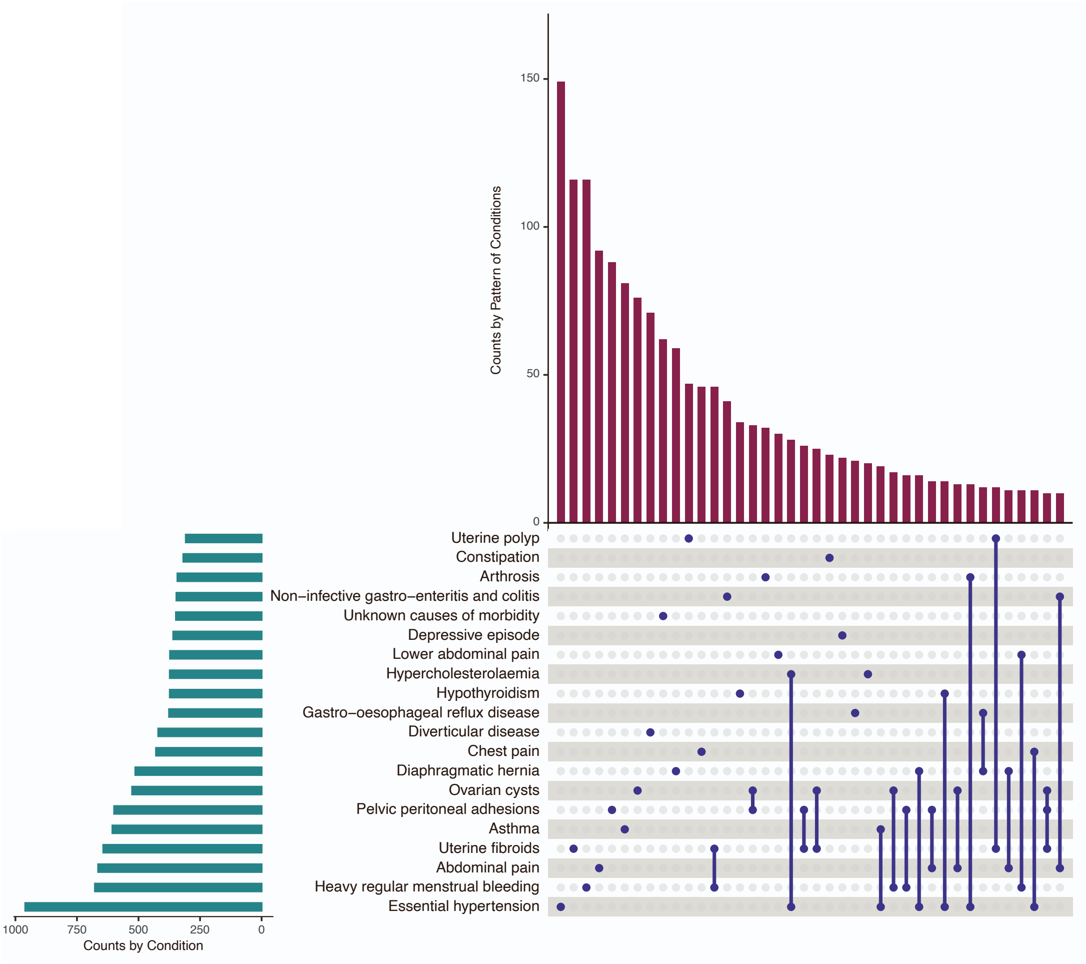

Figure S2. UpSet plot of the most common trait combinations associated with endometriosis in 5,432 endometriosis cases in the UK Biobank. Essential hypertension: I10 Essential (primary) hypertension; Heavy regular menstrual bleeding: N92.0 Excessive and frequent menstruation with regular cycle; Abdominal pain: R10.4 Other and unspecified abdominal pain; Uterine fibroids: D25.9 Leiomyoma of uterus, unspecified; Asthma: J45.9 Asthma, unspecified; Pelvic peritoneal adhesions: N73.6 Female pelvic peritoneal adhesions; Ovarian cysts: N83.2 Other and unspecified ovarian cysts; Diaphragmatic hernia: K44.9 Diaphragmatic hernia without obstruction or gangrene; Chest pain: R07.4 Chest pain, unspecified; Diverticular disease: K57.3 Diverticular disease of large intestine without perforation or abscess; Gastro-oesophageal reflux disease: K21.9 Gastro−oesophageal reflux disease without oesophagitis; Hypothyroidism: E03.9 Hypothyroidism, unspecified; Hypercholesterolaemia: E78.0 Pure hypercholesterolaemia; Lower abdominal pain: R10.3 Pain localised to other parts of lower abdomen; Depressive episode: F32.9 Depressive episode, unspecified; Unknown causes of morbidity: R69 Unknown and unspecified causes of morbidity; Non−infective gastro−enteritis and colitis : K52.9 Non−infective gastro−enteritis and colitis, unspecified; Arthrosis: M19.9 Arthrosis; Constipation: K59.0 Constipation; Uterine polyp: N84.0 Polyp of corpus uteri.

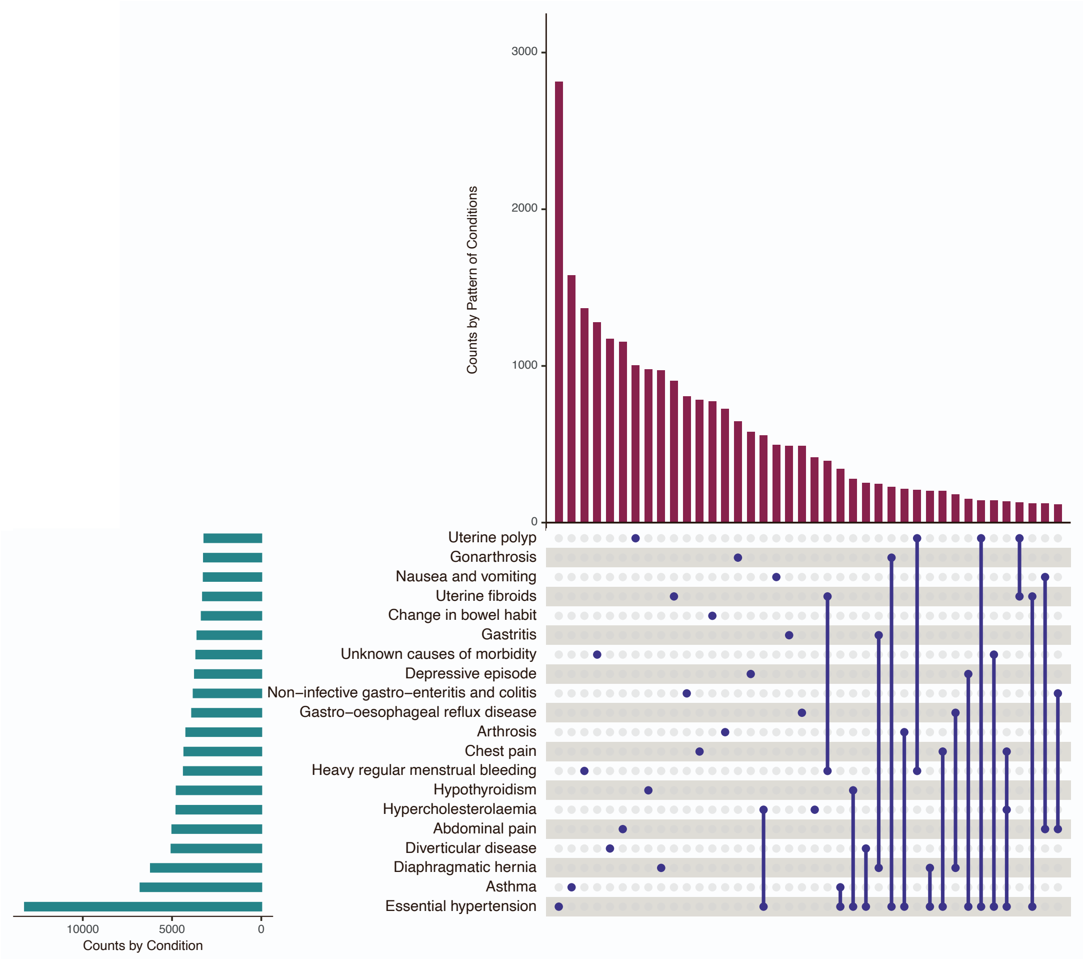

Figure S3. UpSet plot of most common trait combinations associated with endometriosis in 92,344 endometriosis controls in the UK Biobank. Essential hypertension: I10 Essential (primary) hypertension; Asthma: J45.9 Asthma, unspecified; Diaphragmatic hernia: K44.9 Diaphragmatic hernia without obstruction or gangrene; Diverticular disease: K57.3 Diverticular disease of large intestine without perforation or abscess; Abdominal pain: R10.4 Other and unspecified abdominal pain; Hypercholesterolaemia: E78.0 Pure hypercholesterolaemia; Hypothyroidism: E03.9 Hypothyroidism, unspecified; Heavy regular menstrual bleeding: N92.0 Excessive and frequent menstruation with regular cycle; Arthrosis: M19.9 Arthrosis; Chest pain: R07.4 Chest pain, unspecified; Gastro–oesophageal reflux disease: K21.9 Gastro–oesophageal reflux disease without oesophagitis; Non–infective gastro–enteritis and colitis: K52.9 Non–infective gastro–enteritis and colitis, unspecified; Depressive episode: F32.9 Depressive episode, unspecified; Unknown causes of morbidity: R69 Unknown and unspecified causes of morbidity; Gastritis: K29.7 Gastritis, unspecified; Change in bowel habit: R19.4 Change in bowel habit; Uterine fibroids: D25.9 Leiomyoma of uterus, unspecified; Nausea and vomiting: R11 Nausea and vomiting; Gonarthrosis: M17.9 Gonarthrosis, unspecified; Uterine polyp: N84.0 Polyp of corpus uteri.

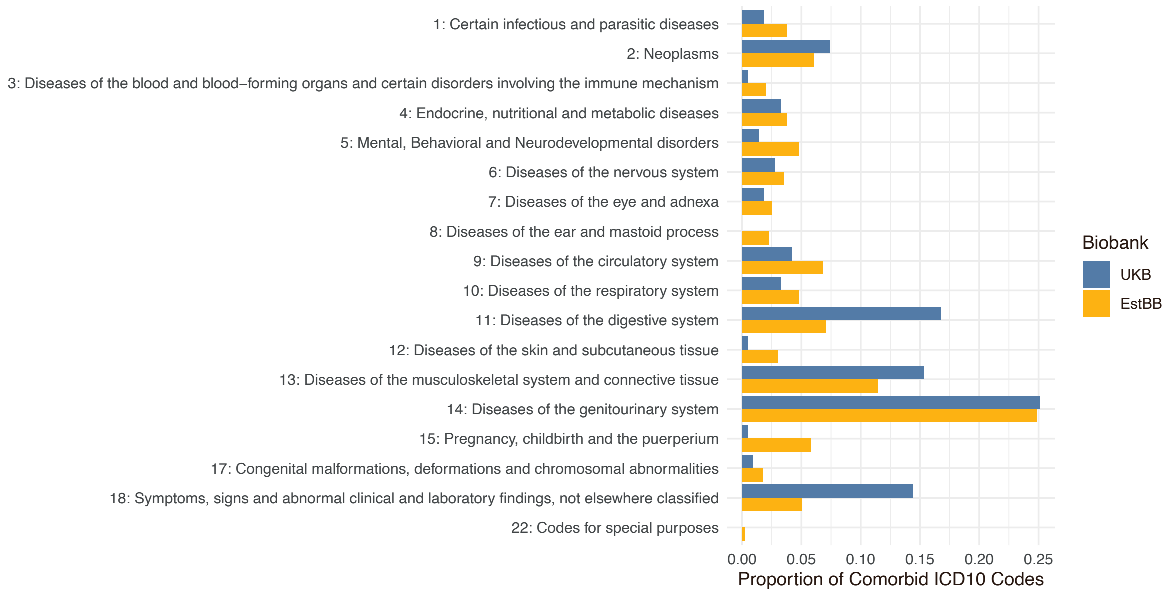

Figure S4. Chapter distribution of ICD10 codes identified as comorbid with endometriosis by biobank. EstBB = Estonian Biobank, UKB = UK Biobank.

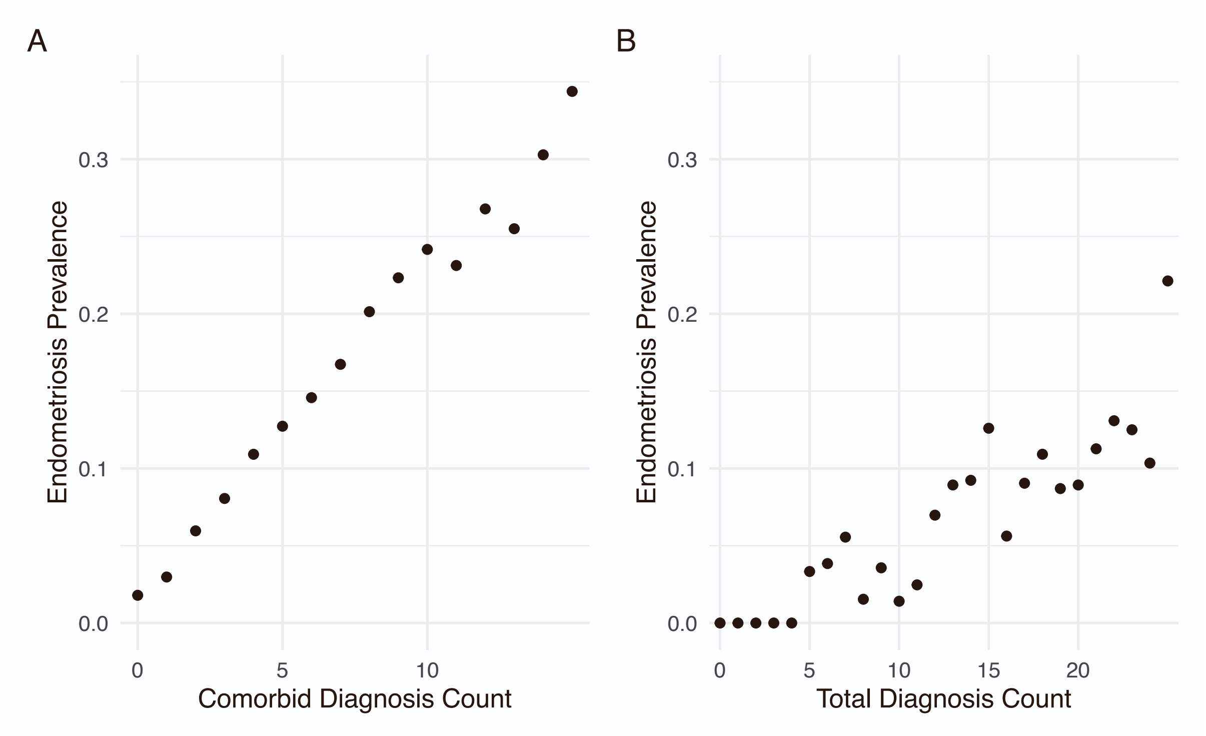

Figure S5. Endometriosis prevalence by comorbidity burden in 3,824 unrelated European ancestry endometriosis cases and 15,296 age matched female controls from the Estonian Biobank. A. Known endometriosis comorbidities (identified in UKB) only. B. All diagnosed conditions.

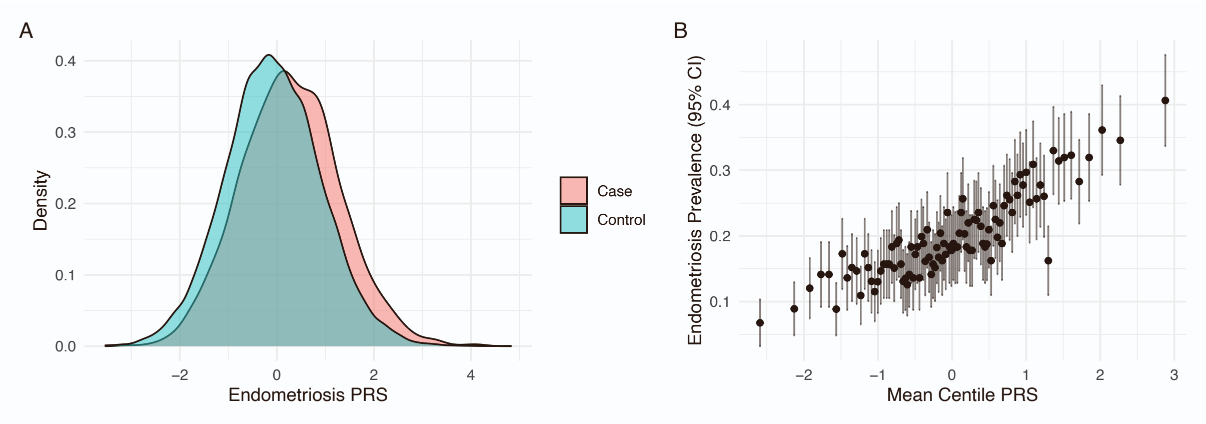

Figure S6. Density plot of endometriosis polygenic risk scores in 3,824 endometriosis cases and 15,296 female controls (unrelated European) in the Estonian Biobank. B. Odds ratio (95% CI) of endometriosis by endometriosis PRS centile compared to individuals in the 50th centile. The mean PRS of each centile is plotted on the x axis.
