# Supplementary material for: Interaction between genetic risk and comorbid conditions in endometriosis
Source: HGG Adv. 2025 May 13;6(3):100456. doi: 10.1016/j.xhgg.2025.100456 (PMC12159439; doi:10.1016/j.xhgg.2025.100456)
Supplement: Document S2. Article plus supplemental information [file mmc3.pdf]

HGG
Advances

ARTICLE

Interaction between genetic risk and comorbid conditions in endometriosis

Isabelle M. McGrath,[1,2,5,*] Valentina Rukins,[3] Triin Laisk,[3] Estonian Biobank Research Team,[3] Sally Mortlock,[1,4] and Grant W. Montgomery[1]

Summary

Endometriosis is a complex disease, and many genetic and environmental risk factors contribute to disease risk. The genetic risk of endometriosis has been well characterized in genome-wide association studies. While few physiological risk factors are known, endometriosis is associated with many comorbid disorders. This study examines the interplay between genetic risk factors, comorbid disorders, and endometriosis. Genetic and health record data from the UK Biobank (5,432 cases; 92,344 controls) and Estonian Biobank (3,824 cases; 15,296 controls) was used to estimate the correlation between comorbidity burden, endometriosis and genetic risk, and to estimate the interactive effects between endometriosis polygenic risk score (PRS) and diagnosis of prevalent comorbidities (uterine fibroids, heavy menstrual bleeding, dysmenorrhea, irritable bowel syndrome, diverticular disease, and asthma) on endometriosis prevalence. The comorbidity burden was significantly higher in endometriosis cases and was positively correlated with endometriosis PRS in women without endometriosis but negatively correlated in women with endometriosis. The absolute increase in endometriosis prevalence conveyed by the presence of several comorbidities (uterine fibroids, heavy menstrual bleeding, dysmenorrhea) was greater in individuals with a high endometriosis PRS compared to a low endometriosis PRS. These findings, consistent across two biobanks, highlight significant interactions between polygenic risk for endometriosis and the diagnosed comorbidities in endometriosis susceptibility that have implications for understanding the underlying mechanisms contributing to disease risk.

Introduction

Endometriosis is a common gynecological condition characterized by the growth of endometrial-like tissue outside of the uterus. Emerging evidence suggests that endometriosis is a systemic disease associated with an extensive array of comorbid conditions across multiple biological systems. The comorbidities affect quality of life, complicate diagnosis due to overlapping symptoms, serve as risk factors that aid in diagnosis, and can both be influenced by and influence the outcome of treatment plans. Both genetic risk and co-occurring conditions contribute to an individual's risk of being diagnosed with endometriosis. The association of endometriosis with its comorbidities and the association of endometriosis with genetic risk factors has been well characterized. Endometriosis is genetically correlated with many of its comorbidities, many genetic risk variants are shared, and some causal relationships have been identified using Mendelian randomization (e.g., endometriosis increasing risk of some histotypes of ovarian cancer).[1–4] However, the interplay between endometriosis comorbidities and genetic risk factors on endometriosis diagnosis remains to be investigated.

An individual's genetic liability for endometriosis can be summarized into a quantitative score, known as a polygenic risk score (PRS). This score is calculated using SNP effects determined from studying the genetic differences between large cohorts of endometriosis cases and controls, in a genome-wide association study (GWAS). The most recent GWAS for endometriosis revealed 42 loci and 49 independent signals associated with endometriosis.[4] Collectively, the 49 genome-wide significant endometriosis SNPs explain 1.98% of the variance in endometriosis (h^2_{SNP}), which increases to 5.01% in individuals with severe disease (stage III/IV).[4] When considering all common genotyped SNPs, the variance explained increases to 26%.[5] While at present the endometriosis PRS in isolation shows limited utility in accurately predicting endometriosis, there is no doubt genetic variants contribute to the risk of developing endometriosis. The possible interaction of the polygenic risk of endometriosis with the diagnosis of other conditions may provide insights into better strategies to predict endometriosis and the biological underpinnings of the disease.

The risk conveyed by many environmental/physiological risk factors has been identified as being modulated by the genetic risk using a variety of approaches for many complex conditions, including coronary artery disease (CAD), adiposity and metabolic syndrome, and depression.[6–11] For example, the absolute increase in the prevalence of CAD upon diagnosis of diabetes, a risk factor for CAD, was 2.7 times greater in individuals with a CAD PRS in the top 10% of scores as opposed to the lowest 10%.[6] Such interactions have not been investigated for endometriosis.

[1]The Institute for Molecular Bioscience, The University of Queensland, Brisbane, 4072 QLD, Australia; [2]Department of Psychiatry, University of Oxford, Oxford, UK; [3]Estonian Genome Center, Institute of Genomics, University of Tartu, 51010 Tartu linn, Estonia; [4]The Australian Women and Girls' Health Research Centre, The University of Queensland, Brisbane, 4072 QLD, Australia
[5]Lead contact
*Correspondence: isabelle.mcgrath@uq.edu.au
https://doi.org/10.1016/j.xhgg.2025.100456.
© 2025 Published by Elsevier Inc. on behalf of American Society of Human Genetics.
This is an open access article under the CC BY-NC-ND license (http://creativecommons.org/licenses/by-nc-nd/4.0/).

This study aims to elucidate the contribution of comorbidity burden to endometriosis risk, the correlation between comorbidity burden and endometriosis polygenic risk, and the interaction between diagnosis of several commonly occurring comorbidities and endometriosis polygenic risk.

Subjects and methods

Cohort selection

The UK Biobank (UKB) was used as the discovery cohort. Unrelated European females with age-matched endometriosis cases (5,432) and controls (92,344) were selected (relatedness defined using genetic information; genetic relationship matrix < 0.05). Endometriosis cases included self-report, primary care, and hospital-diagnosed cases, as previously described.[3] The Estonian Biobank (EstBB) was used to validate the findings.[12] Unrelated European females with age-matched endometriosis cases (3,824) and controls (15,296) were selected for analysis in the EstBB (relatedness defined using genetic information; PI_HAT <0.05). The same criteria used to define the UKB cases and controls were used in the EstBB, although only medical records were utilized as self-reported endometriosis was not recorded.

This study employed data from the UKB and summary statistics from additional sources and were approved by the UKB and the human research ethics committee of The University of Queensland (Project 2020/HE002852). All EstBB participants signed a broad informed consent form. Analyses were carried out under ethical approval 1.1–12/624 from the Estonian Committee on Bioethics and Human Research (Estonian Ministry of Social Affairs), using data according to release application T32 6–7/GI/32219 from the EstBB.

PRS calculation

GWAS summary statistics for endometriosis were generated by meta-analyzing the European subset of the Sapkota et al. (2017) meta-analysis (14,926 cases; 189,715 controls) of endometriosis[13] with the endometriosis GWAS summary statistics obtained from FinnGen Release 8 (13,456 cases; 100,663 controls), as described by McGrath et al.[2] Summary statistics underwent quality control. In the case of duplicate SNPs, the SNP with the lowest p value was selected, and the minor allele frequencies were restricted to >1%. Before computation of PRS, GWAS summary statistics were adjusted with SBayesR to improve PRS prediction, as implemented in GCTB version 2.02. The SNP sample size was imputed with –impute-n. The major histocompatibility complex region was excluded with –exclude-mhc (chr6:28-34 Mb). The PRS for endometriosis was calculated in both the UKB and EstBB datasets using the score function in plink1.9 and plink2, respectively. The PRS was adjusted to Z score in both cohorts.

A second set of PRSs was calculated in the same manner using the endometriosis GWAS summary statistics conditioning for uterine fibroids. The endometriosis GWAS summary statistics from the meta-analysis were adjusted for genetic effects of uterine fibroids using uterine fibroids GWAS summary statistics from Gallagher et al.[14] with mtCOJO as implemented in GCTA version 1.94.1. QIMRHCS was used as the linkage disequilibrium reference sample.[13] SNP effects were reweighted with SBayesR, as above.

Comorbidity search

In both the UKB and EstBB cohorts, any 5-character *International Classification of Diseases, Tenth Revision* (ICD10) codes assigned to the participants were simplified to 4-character codes, and then all ICD10 codes were tested for association with endometriosis using the chi-squared test or Fisher's exact test, where appropriate. Here, a comorbidity of endometriosis is defined as a condition with which someone with endometriosis is likely to be diagnosed at any point during their lifetime, and the condition does not necessarily need to occur concurrent with endometriosis. In the UKB cohort, 287 codes were associated with endometriosis at a false discovery rate of 5% (Table S1). In the EstBB dataset, 455 codes were associated with endometriosis at a false discovery rate of 5% (Table S2).

Trait selection and counting

Individual-level data were filtered to exclude ICD10 diagnoses related to external factors (codes in chapters 19 [Injury, Poisoning, and Certain Other Consequences of External Causes], 20 [External Causes of Morbidity and Mortality], and 21 [Factors Influencing Health Status and Contact with Health Services]) and endometriosis codes N80.1–N80.9. Counting metrics are outlined in Figure 1. As performed for the comorbidity search, any 5-character codes were simplified to 4-character codes. To assess disease burden, each participant's count of all diagnosed conditions ("total diagnoses") and comorbid diagnosed conditions ("comorbid diagnoses") were determined. Comorbid conditions included codes identified as comorbid with endometriosis in the comorbidity search after the exclusion of codes with significant decreased risk. Most codes for which endometriosis cases had significant decreased risk related to pregnancy. To avoid inflation from circular analysis, comorbidity codes identified in the alternate biobank were used (i.e., codes identified as comorbid with endometriosis in the EstBB were counted as comorbidities in the UKB and vice versa). To reduce granularity of the data, as ICD10 codes are highly specific, the results presented here are the count of unique 3-character ICD10 diagnoses. The results pertaining to the count of unique 4-character diagnoses are presented in the supplemental information. We note that because some ICD10 codes in the biobanks have only 3-characters in their nonsimplified form, the 4-character ICD10 codes include some 3-character codes; therefore, "4-character" and "3-character" refer to the maximum resolution of the ICD10 codes permitted in that analysis. The comorbid diagnosis count and total diagnosis count were also assessed chapter-wise and in a leave-one-chapter-out manner, whereby the count of unique 3-character ICD10 codes was found.

Individuals in the cohorts were also ascertained for diagnosis of several "index" comorbidities (selected commonly diagnosed comorbidities: asthma, diverticular disease, irritable bowel syndrome, uterine fibroids, dysmenorrhea, and heavy menstrual bleeding) using ICD10, primary care, and self-report information, where available. The definitions of these index comorbidities were as follows: asthma—self-report, primary care, ICD10 (J45.9 Asthma, unspecified, J45.0 Predominantly allergic asthma, J45.1 Nonallergic asthma, J45.8 Mixed asthma); diverticular disease—self-report (diverticular disease/diverticulitis), primary care, ICD10 (K57.X); irritable bowel syndrome—self-report, primary care, (K58.X); uterine fibroids—self-report, ICD10 (D25.9 Leiomyoma of uterus, unspecified, D25.1 Intramural leiomyoma of uterus, D25.2 Subserosal leiomyoma of uterus, D25.0 Submucous

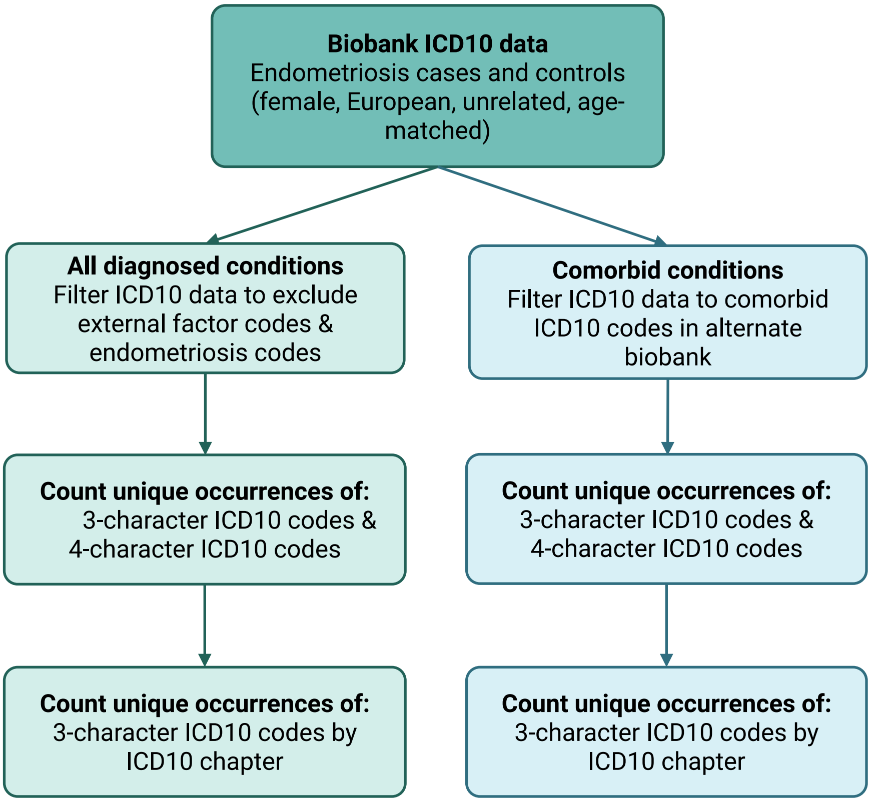

Figure 1. ICD10 counting metrics
Created in BioRender.com.

leiomyoma of uterus); dysmenorrhea—self-report, ICD10 (N94.6 Dysmenorrhea, unspecified, N94.4 Primary dysmenorrhea, N94.5 Secondary dysmenorrhea); and heavy menstrual bleeding—ICD10 (N92.0 Excessive and frequent menstruation with regular cycle), noncancer self-report: "menorrhagia (unknown cause)."

Statistical analysis

The efficacy of the endometriosis PRS in the UKB was tested by calculating the area under the curve (AUC) using the pROC package. Individuals were stratified into deciles and centiles based on their endometriosis PRS. The odds ratio of endometriosis in each centile was determined, using the 50th centile as the reference. Endometriosis prevalence by diagnosis burden was determined by grouping individuals by their unique 3-character ICD10 diagnosis count and determining the prevalence of endometriosis within each group. The relationship between endometriosis PRS and comorbidity burden was evaluated in a linear model, whereby the comorbidity burden was modeled by the endometriosis PRS and endometriosis status, including an interactive term between PRS and endometriosis, for each trait-counting metric. To assess whether trends were consistent across biological trait groupings, this was repeated in an ICD10 chapter-wise manner using the 3-character ICD10 code counts. As an additional sensitivity test, leave-one-chapter-out counts were also calculated using the 3-character ICD10 code counts.

An interactive effect can be measured in two scales: the additive scale and the multiplicative scale. A positive additive interactive effect exists when the effect of two risk factors together exceeds the sum of the effects of the two risk factors considered separately.[15] A positive multiplicative interactive effect exists when the effect of two risk factors together exceeds the product of the effects of the two risk factors considered separately.[15] The additive scale is more useful in determining the utility of public health measures, although it is recommended that both terms be estimated.[15] An interactive effect on the additive scale was assessed by comparing the absolute increase in prevalence of endometriosis upon comorbidity diagnosis across PRS deciles. An interactive effect between endometriosis PRS and comorbidity presence (using the six index comorbidities) on the multiplicative scale was assessed using a logistic regression model, modeling endometriosis presence by endometriosis PRS, index comorbidity presence, and an interactive effect of the endometriosis PRS and index comorbidity presence.

Statistical tests were conducted with individual-level data; however, aggregated (decile/centile-level) data have been used for plotting for readability. Analysis was conducted in R version 4.1.3. All statistical analyses were repeated in the EstBB. The difference in the proportion of comorbid codes identified within a chapter between biobanks was compared with the prop.test in R.

Results

Endometriosis comorbidities in the UKB and EstBB

The UKB dataset included 5,432 unrelated European ancestry endometriosis cases and 92,344 female controls. The mean age of endometriosis diagnosis was 39.2 years. Diagnostic data from hospital-linked records used in this analysis are from either 1981–2021 or 1999–2021 (depending on the region of residence in the UK[16]). There were 287 four-character ICD10 codes associated with an endometriosis diagnosis (Table S1). This reduced to 215 four-character codes after excluding codes related to external factors. Prevalent traits significantly associated with endometriosis included hypertension, menstrual disturbances, uterine fibroids, and asthma (Figure S1). When considering the prevalence of every diagnostic combination within the significantly associated set, a diagnosis of only primary hypertension was most common for both endometriosis cases and controls (Figures S2 and S3).

The EstBB cohort included 3,824 endometriosis cases and 15,296 controls with electronic health record data from 2004.[12] The average age of endometriosis diagnosis was 35.5 years. In the EstBB, 455 ICD10 codes were significantly associated with endometriosis (Table S2). After excluding codes relating to external factors and those more frequent in controls, 394 codes remained. Within the filtered ICD10 code sets, 91 were identified in both

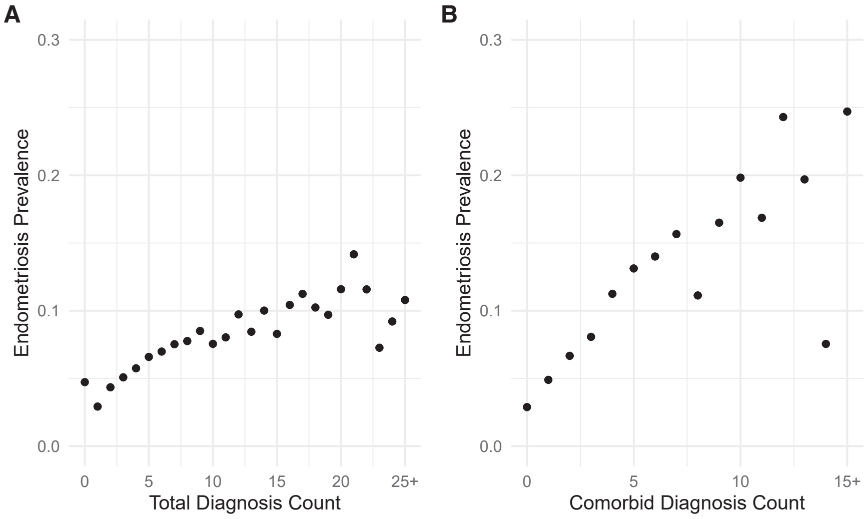

Figure 2. Endometriosis prevalence by comorbidity burden in 5,432 unrelated European ancestry endometriosis cases and 92,344 age-matched female controls from the UKB
(A) All diagnosed conditions.
(B) Known endometriosis comorbidities (identified in EstBB) only.

the UKB and EstBB, 124 were identified only in the UKB, and 303 were identified only in the EstBB. In both biobanks, approximately 25% of the comorbid codes were classified as genitourinary diagnoses. Considering the distribution of filtered comorbid codes across chapters between the two biobanks, a significantly greater proportion of comorbid codes were in chapter 11 (Diseases of the Digestive System, p = 3.59e−4) and chapter 18 (Symptoms, Signs, Abnormal Clinical and Laboratory Findings, p = 1.31e−4) in the UKB compared to the EstBB, while chapter 15 (Pregnancy, Childbirth, and the Puerperium, p = 2.38e−3) had a significantly greater proportion of comorbid codes in the EstBB compared to the UKB (Figure S4).

The prevalence of endometriosis increased with comorbidity burden when considering both known endometriosis comorbidities and all conditions diagnosed in the cohort, in both the UKB (Figure 2) and EstBB (Figure S5). Given that the control cohorts were selected to match the cases by age, the reported prevalence of endometriosis should not be considered the population prevalence in either cohort; instead, the result of interest is the relative differences between the groupings.

Polygenic risk for endometriosis

The PRS for endometriosis was calculated in the age-matched endometriosis case-control cohorts. Endometriosis cases had a higher mean PRS than endometriosis controls, corresponding to an AUC-receiver operating characteristic of 0.61 in the UKB (Figure 3A). Using individuals in the 50th centile as the reference group, individuals in the 1st centile had an odds ratio of 0.38 of being diagnosed with endometriosis, whereas individuals in the 100th centile had an odds ratio of 3.67 (Figure 3B).

In the EstBB, the AUC of the PRS was 0.60. The odds ratio of endometriosis in the 1st PRS centile was 0.32, whereas the odds ratio of endometriosis within individuals in the top 1% of PRS was 3.04 (Figure S6).

Correlation between comorbidity burden and endometriosis PRS

In the UKB, endometriosis cases had a greater average comorbidity burden than endometriosis controls (Figure 4A). The average burden of all diagnosed 3-character ICD10 codes were as follows: case mean = 7.41, control mean = 5.06, t test p = 3.71e−114. The average burden of the known endometriosis comorbidity 3-character ICD10 codes were: case mean = 2.66, control mean = 1.38, t test p = 1.25e−238). The increased comorbidity burden of endometriosis cases compared to endometriosis controls was consistently observed irrespective of the diagnosis count approach in the UKB and EstBB (Table S3).

The relationship between comorbidity burden and endometriosis PRS was considered in endometriosis cases and controls. In individuals without an endometriosis diagnosis in the UKB, a positive relationship was observed between endometriosis PRS and comorbidity burden, whereas in individuals with an endometriosis diagnosis, there was a negative relationship between endometriosis PRS and co-occurring trait burden (Figure 4B). The effect of the negative interaction between endometriosis status and PRS on comorbid diagnosis burden was statistically significant in a linear regression model in the UKB (interaction: −0.155 ± 0.028, p = 2.01e−8) (Table S4). The interactive term was also negative when considering total diagnoses (Table S4). In the EstBB, the interactive term was consistently negative but only reached significance in the total diagnosis analysis (Table S4). In a sensitivity analysis, where biological groupings of codes were withheld from the trait count one by one, the negative interaction term remained significant for the total diagnosis counts in both the UKB and EstBB, and for the comorbidity diagnosis counts in the UKB (Table S5). Chapter-specific comorbidity burdens were also calculated and assessed in relation to endometriosis PRS. The interactive term derived from the chapter-specific comorbidity burdens were likewise generally negative in the UKB and EstBB (Table S6). One chapter in the UKB had a significant positive interactive term when considering the comorbid diagnoses: chapter 2 (Neoplasms) (PRS × Endometriosis = 0.015 ± 0.004, p = 5.05e−4). There were two results with a significant positive interactive term in the EstBB: comorbid conditions in chapter 2 (Neoplasms) (PRS × Endometriosis = 0.033 ± 0.012, p = 6.41e−3) and 14

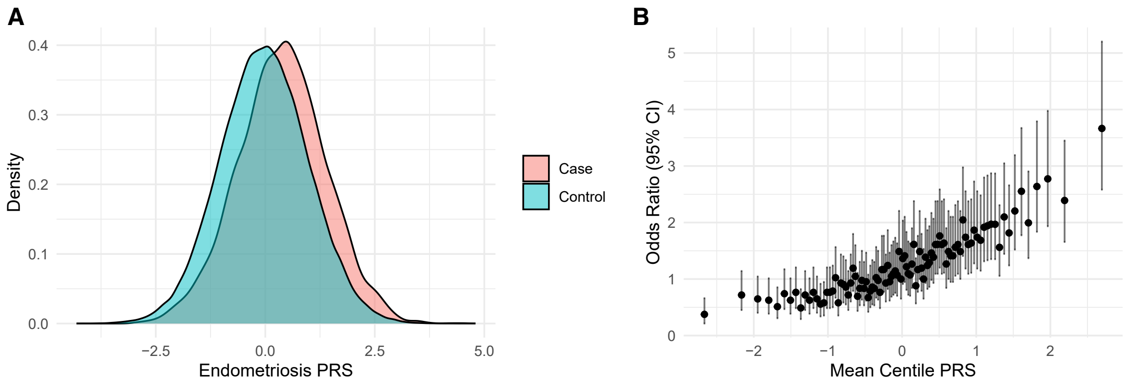

Figure 3. Endometriosis polygenic risk scores and endometriosis in the UKB
(A) Density plot of endometriosis polygenic risk scores in 5,432 endometriosis cases and 92,344 female controls (unrelated European) in the UKB.
(B) Odds ratio (95% confidence interval [CI]) of endometriosis by endometriosis PRS centile compared to individuals in the 50th centile. The mean PRS of each centile is plotted on the *x* axis.

(Diseases of the Genitourinary System) (PRS × Endometriosis = 0.087 ± 0.036, $p = 0.015$).

The interaction effect between comorbidity diagnosis and endometriosis PRS on endometriosis risk

An interactive effect between endometriosis PRS and diagnosis of several commonly co-occurring endometriosis comorbidities (uterine fibroids, dysmenorrhea, heavy menstrual bleeding, diverticular disease, asthma, and irritable bowel syndrome) was assessed (Figure 5; Table S7). In the 1st endometriosis PRS decile in the UKB, the absolute prevalence of endometriosis was 5.67% greater in individuals with uterine fibroids compared to those without uterine fibroids. This increases to 15.57% in the 10th endometriosis PRS decile (Table S7). Very similar statistics were seen in the EstBB, where the absolute prevalence of endometriosis was 5.97% greater in the 1st decile and 15.61% greater in the 10th decile upon uterine fibroids diagnosis (Table S7). Therefore, the absolute prevalence increase in endometriosis upon diagnosis of uterine fibroids is approximately 2.7 times greater in the 10th endometriosis PRS decile than the 1st PRS decile. This additive interactive effect for uterine fibroids with endometriosis PRS was retained when adjusting the GWAS summary statistics used for calculating the endometriosis PRS for uterine fibroids (Table S8). Similar suggestions of an additive interactive effect were observed for the other investigated conditions (e.g., diverticular disease with a 2.5 times and heavy menstrual bleeding with a 2.1 times increase in absolute prevalence between 1st and 10th PRS deciles) (Figure 5; Table S7). On the multiplicative scale, nominally significant interactions between endometriosis PRS and comorbidity diagnosis were noted: there was a significant negative interactive effect for asthma ($p = 0.016$) and irritable bowel syndrome ($p = 0.018$) in the UKB and for heavy menstrual bleeding in the EstBB ($p = 0.02$) (Table S9).

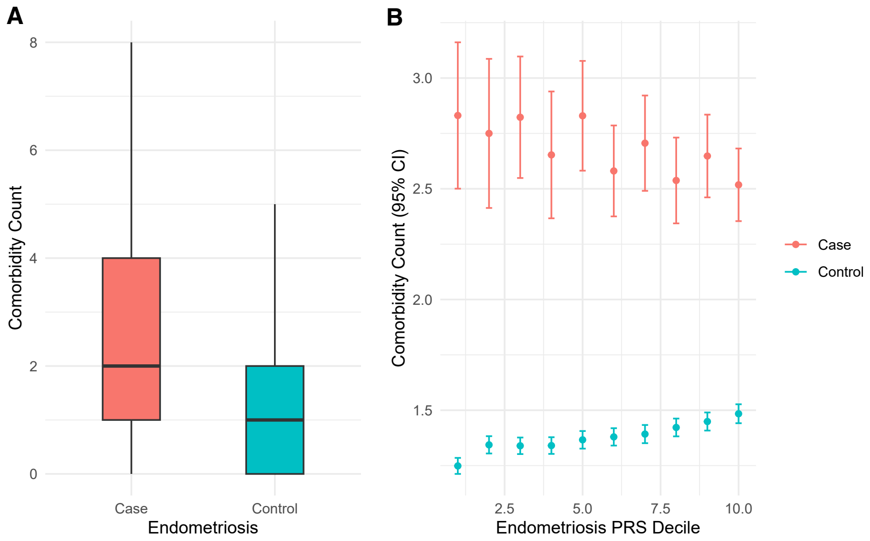

Figure 4. Comorbidity burden of endometriosis cases (5,432) and age matched controls (92,344) in the UKB (unrelated European females)
(A) Boxplot of comorbidity count distributions in the UKB in endometriosis cases and controls. Outliers are excluded from the plot.
(B) Comorbidity count by endometriosis PRS decile, stratified by endometriosis status. The mean comorbidity count (95% CI) of each PRS decile is plotted separately in cases and controls. Comorbidity count is the count of unique 3-character ICD10 codes previously determined as comorbid with endometriosis.

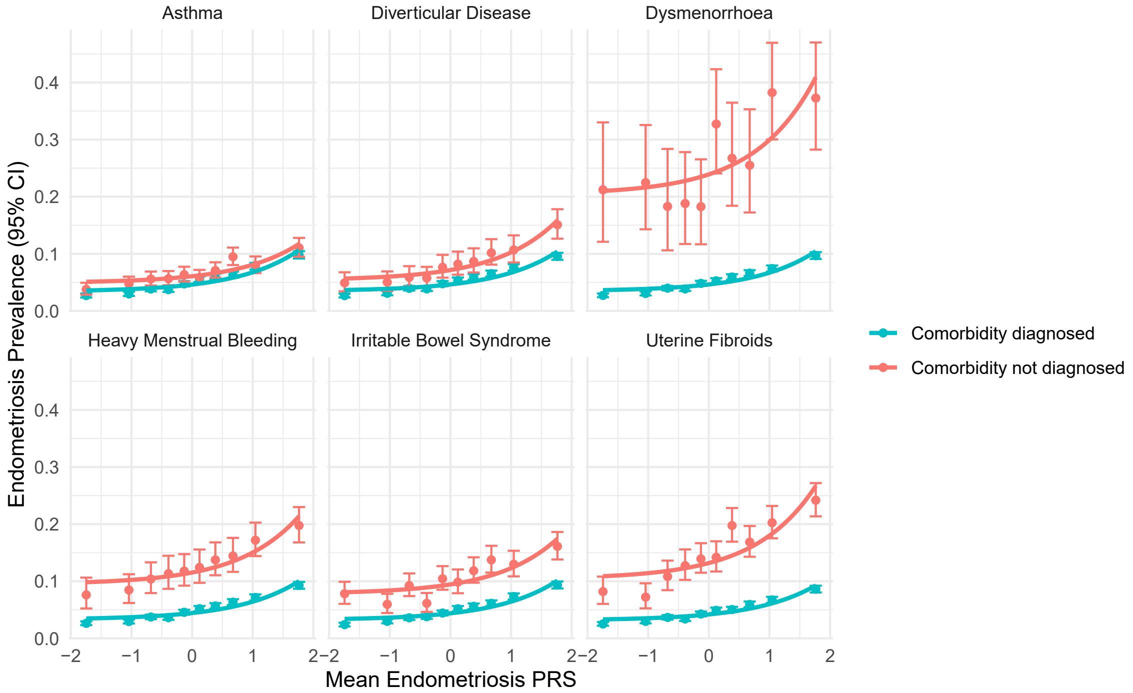

Figure 5. Endometriosis prevalence by endometriosis PRS decile (mean value ± 95% CI), stratified by comorbidity diagnosis in 5,432 endometriosis cases and 92,344 age-matched controls in the UKB (unrelated European females)

Discussion

Understanding how risk factors interact to cause disease can provide insights into disease developmental pathways, enhance risk stratification, and inform risk-reducing strategies. Endometriosis has poorly understood etiology, few physiological risk factors, and limited management approaches. In this study, polygenic risk of endometriosis and diagnosis of its comorbidities were found to have a complex relationship in the two investigated cohorts, the UKB and the EstBB. Overall, these findings indicate that a substantial comorbidity burden is associated with endometriosis and that the comorbidities and its polygenic risk are related factors.

Women with endometriosis were identified to have a greater burden of comorbidities than women without an endometriosis diagnosis. While an increased diagnostic burden is not likely to be unique to endometriosis, quantification of the elevated comorbidity burden is important for estimating the total cost and impact of endometriosis and provides additional evidence for the need for a multi-disciplinary model of care. This elevated burden could contribute to the heterogeneity in endometriosis, symptoms, and treatment responses and therefore has important implications for diagnosis and management. Care for endometriosis is typically framed around the gynecological system; however, as thought for many other conditions, management programs addressing the individual multimorbidity may be more effective.[17,18] The older age of the UKB participants allowed for a comprehensive, lifelong comorbidity analysis. Conditions typically occurring later in life were correlated with endometriosis; hypertension was the most commonly occurring comorbidity, although it showed only a slightly elevated prevalence between endometriosis cases (17.6%) and controls (14.3%). The association of later-onset conditions with endometriosis is unlikely to be fully accounted for by endometriosis-motivated healthcare exposure. Therefore, a diagnosis of endometriosis may have lifelong effects.

In this study, we found the correlation of comorbidity burden with endometriosis polygenic risk to be dependent on endometriosis diagnosis. In individuals without an endometriosis diagnosis, higher endometriosis polygenic risk was correlated with increased number of non-endometriosis diagnoses. Endometriosis shares a genetic architecture with many other conditions[1–4]; therefore, the pleiotropic nature of the endometriosis risk variants means that in those with a high endometriosis polygenic risk but not evidence of endometriosis, other conditions are more likely to develop. In individuals with an endometriosis diagnosis, endometriosis polygenic risk was negatively correlated with comorbidity burden. The negative interaction effect between PRS and endometriosis status on comorbidity burden was robust in sensitivity analyses where biological groupings of traits were omitted from the burden estimation, suggesting one biological group of traits is not likely to drive this relationship. While trends in both cases and controls are moderate at best, they may point to important biological mechanisms

critical for understanding endometriosis and managing the condition.

An inverse association between comorbidity burden and PRS in cases is expected in a threshold model of disease whereby disease develops after a threshold is surpassed through additive small effects of many genetic and environmental risk factors. The comorbidities could be such environmental risk factors (acknowledging the comorbidities have overlapping genetic architectures with endometriosis and therefore are not purely environmental). The threshold model is thought to explain endometriosis.[19] According to the threshold model, individuals beyond the threshold (i.e., with disease) who have a high polygenic risk will on average have fewer other risk factors than individuals with a low polygenic risk, as fewer were needed to cross the disease threshold (i.e., develop the disease). The relationship between polygenic risk and comorbidities in endometriosis cases reflects this trend, suggesting that the comorbidities contribute to endometriosis risk. Previously published Mendelian randomization analyses have identified only a few robust causal relationships between endometriosis and its comorbidities.[1–3,20–23] These previous non-causal findings could be explained by insufficient power to detect causal associations; a contribution of comorbidity burden, rather than a specific comorbidity, to endometriosis risk; or the causal effect of the comorbidities acting through an underlying biological pathway that is not well captured by the entire genetic architecture of the comorbidity. Indeed, genetic variants are shared between endometriosis and its comorbidities,[3] suggesting that the comorbidities contribute to endometriosis risk in a direct or indirect manner. The relationship between a condition's PRS and comorbidity burden has not been studied extensively for many other complex conditions, although a recent preprint found a similar association: in individuals with atrial fibrillation, the genetic risk to atrial fibrillation was highest in a cluster of individuals with few comorbidities.[24] Other contributing factors to this trend are possible, including genetic influences on disease heterogeneity (endometriosis features and comorbidities), which could impact health service usage. There is some evidence for the variable effects of the endometriosis-risk SNPs—they have greater effect sizes in severe disease compared with minimal/mild disease,[13] their burden increases from mild disease to severe disease,[25] and they show variable associations with comorbidities.[3] The presentation of endometriosis in an individual (e.g., lesion characteristics and symptoms) is likely related to the ease of diagnosis, which may influence healthcare-seeking behavior and diagnostic pathways. For example, if there is a long delay between symptom onset and diagnosis, then many conditions may be diagnosed while investigating the cause of the symptoms. Conversely, individuals with rapidly diagnosed endometriosis may be less likely to be diagnosed with some comorbidities, particularly symptoms like dysmenorrhea or heavy menstrual bleeding, due to their overlap with endometriosis itself. Therefore, studying the relationship between comorbidity burden and endometriosis PRS alongside detailed clinical and healthcare utilization data will be invaluable in disentangling these effects.

Chapter-specific analyses indicated that most chapters had a negative interaction effect between PRS and endometriosis diagnosis on comorbidity burden, although there were a few exceptions. Chapter 2 (Neoplasms) had a positive interaction effect between PRS and endometriosis diagnosis on comorbid diagnosis burden in both biobanks. Endometriosis shares germline and somatic mutations with cancers, and a causal effect of endometriosis on several histotypes of ovarian cancer has previously been identified.[20,26–28] Therefore, this synergistic relationship may reflect common effects on cell proliferation and cell-cycle control contributing to the initiation of neoplasms and endometriosis lesions. The interactive term was non-significant when considering all diagnoses in the chapter in both biobanks, suggesting selective associations of endometriosis with cancer types, which is supported by previous genetic studies.[3,29] In the EstBB, there was a positive interaction for comorbid diagnoses in chapter 14 (Diseases of the Genitourinary System). This was not observed in the total diagnoses count analysis for this chapter, or in the UKB, and it should be noted this p value for the association would not have passed multiple testing correction ($p = 0.015$). Nevertheless, severe disease, often characterized by ovarian cysts and deeply infiltrating endometriosis, is likely to involve a higher endometriosis genetic risk burden and therefore more likely to be associated with other genitourinary conditions.

There are several possible causal pathways connecting the comorbidities of endometriosis, the polygenic risk for endometriosis and endometriosis (Figure 6). Interaction analyses suggested that the effects of some endometriosis comorbidities are modulated by the endometriosis PRS. The greatest difference in the increase in the absolute prevalence of endometriosis upon diagnosis of the comorbidity between individuals in the lowest 10% and highest 10% of endometriosis genetic risk was seen for uterine fibroids (5.7% increase in endometriosis risk in the 1st decile, 15.6% increase in the 10th decile). This means the risk conferred by uterine fibroids on endometriosis is dependent on an individual's PRS for endometriosis; the risk of endometriosis in those with uterine fibroids increases with endometriosis PRS. Uterine fibroids, also referred to as uterine leiomyomas, are benign tumors in the myometrium of the uterus. Of the comorbidities studied to date, uterine fibroids have one of the highest genetic correlations with endometriosis ($r_g = 0.39–0.43$), and there is evidence for many loci for a shared causal variant with endometriosis.[3,14] The interactive effect was retained upon adjustment of the PRS for these shared genetic effects, suggesting that there is an underlying pathway of uterine fibroids not dependent on the genetics that overlaps with endometriosis that increases the risk of endometriosis. As uterine fibroids are typically diagnosed

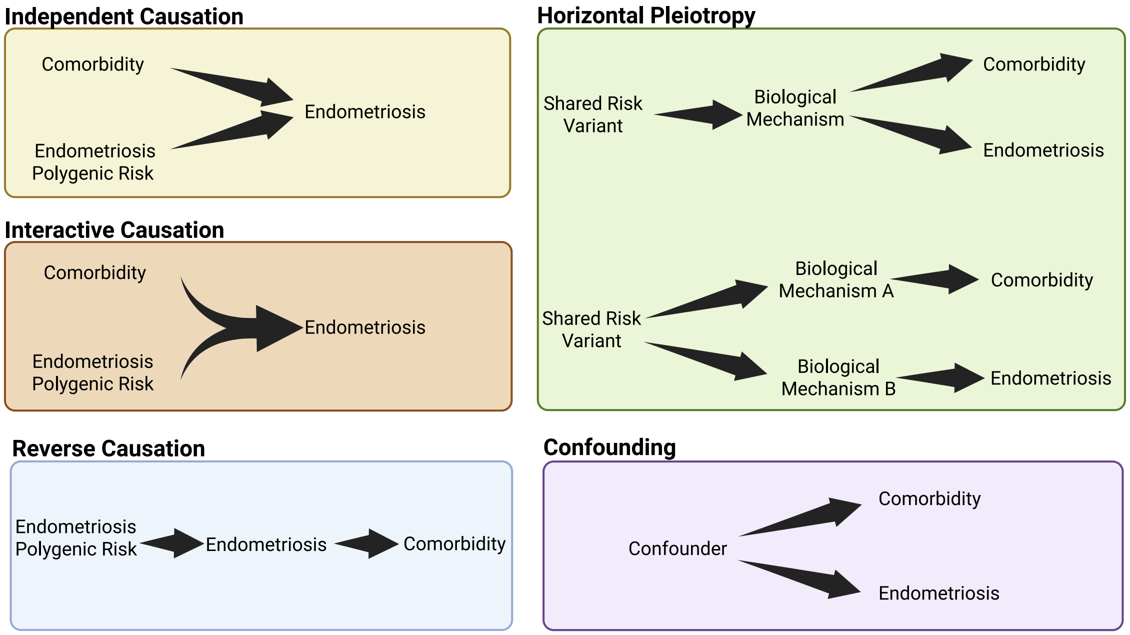

Figure 6. Possible relationships between endometriosis, its comorbidities, and polygenic risk
Here, "causation" is defined as a variable (e.g., endometriosis polygenic risk, comorbidity) increasing the risk of endometriosis. "Reverse causation" is when endometriosis has a risk-increasing effect on the variable. The variable may be related to endometriosis through a non-causal pathway, such as horizontal pleiotropy or confounding.
Created in BioRender.com.

later than endometriosis,[30] this biological mechanism leading to both uterine fibroids and endometriosis may be activated at an earlier stage. While the affected cell type differs from endometriosis, driving pathways are shared (estrogen and progesterone signaling, cell growth).[14] The genetic predisposition to endometriosis has previously been identified as causal for uterine fibroids using Mendelian randomization.[14] The interaction effect of endometriosis PRS with uterine fibroids diagnosis on endometriosis risk provides further evidence that both diseases are driven by a common mechanism. Several antagonistic interactive effects on the multiplicative scale were identified as an artifact of this statistic being dependent on the baseline risk (without-comorbidity risk of endometriosis); individuals with a high endometriosis PRS are still at an increased risk of endometriosis upon diagnosis of any of the six comorbidities analyzed.

Neither the UKB nor EstBB is a lifelong collection of diagnoses for every participant. The EstBB recruited from a younger age (18 years and older), more recently (the majority of the cohort, 150,000 individuals, was recruited in 2018–2019), and has electronic health record data from 2004.[12] Conversely, the UKB recruited participants between 40 and 69 years of age in 2006–2010 and has linked hospital in-patient records dating back to 1981 in Scotland, 1997 in England, and 1999 in Wales. Therefore, there are likely many records from the participants' early adult lives missing from the UKB data, whereas the EstBB participants are comparatively younger and therefore have shorter medical histories. The recent recruitment and younger ages of the EstBB cohort mean that the EstBB may have richer diagnostic data for young adults, whereas the UKB may have richer diagnostic data for older adults. This is reflected in a greater proportion of the comorbid codes of endometriosis in the EstBB belonging to the ICD10 chapter for pregnancy than in the UKB identified comorbid codes.

There are several limitations to this analysis that should be considered in future studies. This analysis was restricted to European cohorts, so generalization of the findings to non-European cohorts may be inappropriate. Furthermore, endometriosis is a highly heterogeneous disease: the location, type, size, and penetration into the tissue can vary. This heterogeneity was not considered in this study, owing to the inability of the ICD10 codes to appropriately capture these features and to the need for even-larger case cohort sizes to allow for powered subset analyses. As symptoms of endometriosis typically emerge in early adulthood, the disease likely develops during one's adolescence. Therefore, studying the comorbidity profiles prior to a diagnosis of endometriosis will be immensely helpful in determining the relationship of the comorbidity burden and the comorbidities themselves with the development of endometriosis. Nevertheless, conditions diagnosed after endometriosis may be endpoints of disrupted biological pathways present prior to endometriosis that could play a role in disease development and are still useful for studying the downstream effects of endometriosis. Lastly, endometriosis has variable symptom severity, is challenging to diagnose, and has long diagnostic delays,

implying that many individuals with the disease may never receive an official diagnosis. Furthermore, the frequency of endometriosis in these biobanks is substantially below current population estimates. The Estonian cohort includes many young individuals who may have yet to seek a diagnosis, and the UKB likely includes many missed diagnoses of endometriosis due to the older age of the cohort (less disease awareness and diagnosis access). The control cohorts used in this study likely include undiagnosed endometriosis cases, so trends in endometriosis controls in this study may be under-estimated.

Endometriosis patients experience a broad range of comorbidities across multiple biological systems. In this study, a substantial comorbidity burden was associated with endometriosis, and a complex relationship between polygenic risk and comorbidity burden was identified. Furthermore, an interactive effect of uterine fibroids with endometriosis PRS on endometriosis risk was discovered. Future research should examine conditions before and after an endometriosis diagnosis using longitudinal and trajectory analyses, incorporating detailed clinical information (e.g., disease grade, site, time from symptom onset to diagnosis), health service use data, and genetic analyses to identify shared etiological factors between endometriosis and its comorbidities. Additionally, cross-trait analyses using large-scale biobank and multi-omics data will help reveal whether conditions co-occur due to shared genetic risk or interacting biological mechanisms, improving our understanding of how genetic predisposition shapes complex disease networks over time. The findings of this study imply that the PRS and the comorbidities are not independent risk-increasing factors, and therefore have implications for creating risk-prediction models, understanding the development of endometriosis, and managing the disease.

Data and code availability

Data utilized in this study can be accessed from the UKB (https://www.ukbiobank.ac.uk/) and the EstBB (https://genomics.ut.ee/en/content/estonian-biobank) per their published data access procedures. Summary data for the FinnGen Endometriosis GWAS are available from their results portal (https://www.finngen.fi/en/access_results). Endometriosis GWAS summary statistics, where they are not publicly available, are available on request. Any additional data supporting the conclusions of this article are included within the article and its supplemental information. Software used in this study is detailed in the subjects and methods section and are available at plink1.9 (https://www.cog-genomics.org/plink/), plink2 (https://www.cog-genomics.org/plink/2.0/), GCTB (https://cnsgenomics.com/software/gctb/#Download), and GCTA (https://yanglab.westlake.edu.cn/software/gcta/#Download).

Acknowledgments

Summary statistics from the endometriosis GWAS used in this study contain data from FinnGen and the International Endometriosis Genomics Consortium (IEGC). We want to acknowledge the participants and investigators of the FinnGen study and the IEGC for making this work possible. This research has been conducted using the UKB resource under application no. 54861. Individual-level phenotype information (including age and ICD10 diagnosis) and genotype data for genetic risk assessment was accessed under application no. 54861. We thank the UKB participants and the UKB research teams for their generous contributions to generating an important research resource. This work uses data provided by patients and collected by the National Health Service (NHS) as part of their care and support. Copyright ©2024, NHS England. Re-used with the permission of the UK Biobank. All rights reserved. We thank the participants of the EstBB for providing valuable resources. Computations of the EstBB data were performed in the High-Performance Computing Center of the University of Tartu. G.W.M. was supported by National Health and Medical Research Council Fellowship GNT1177194. S.M. was supported by Medical Research Future Fund Research grant no. MRF1199785. V.R. was supported by Estonian Research Council grant no. PRG1911. T.L. was supported by Ministry of Education and Research of Estonia grant no. TK214.

Author contributions

I.M.M.: conception, data analysis, result interpretation, and writing and editing. S.M.: editing manuscript, supervision, and results interpretation. G.W.M.: conception, editing manuscript, supervision, and results interpretation. V.R.: data analysis. T.L.: supervision of data analysis.

Declaration of interests

The authors declare no competing interests.

Supplemental information

Supplemental information can be found online at https://doi.org/10.1016/j.xhgg.2025.100456.

Received: January 15, 2025
Accepted: May 9, 2025

References

1. McGrath, I.M., Montgomery, G.W., and Mortlock, S. (2023). Insights from Mendelian randomization and genetic correlation analyses into the relationship between endometriosis and its comorbidities. Hum. Reprod. Update *29*, 655–674. https://doi.org/10.1093/humupd/dmad009.
2. McGrath, I.M., International Endometriosis Genetics Consortium, Montgomery, G.W., and Mortlock, S. (2023). Polygenic risk score phenome-wide association study reveals an association between endometriosis and testosterone. BMC Med. *21*, 482. https://doi.org/10.1186/s12916-023-03184-z.
3. McGrath, I.M., International Endometriosis Genetics Consortium, Montgomery, G.W., and Mortlock, S. (2023). Genomic characterisation of the overlap of endometriosis with 76 comorbidities identifies pleiotropic and causal mechanisms underlying disease risk. Hum. Genet. *142*, 1345–1360. https://doi.org/10.1007/s00439-023-02582-w.
4. Rahmioglu, N., Mortlock, S., Ghiasi, M., Møller, P.L., Stefansdottir, L., Galarneau, G., Turman, C., Danning, R., Law, M.H.,

Sapkota, Y., et al. (2023). The genetic basis of endometriosis and comorbidity with other pain and inflammatory conditions. Nat. Genet. *55*, 423–436. https://doi.org/10.1038/s41588-023-01323-z.

5. Lee, S.H., Harold, D., Nyholt, D.R., Montgomery, G.W., Goddard, M.E., Zondervan, K.T., Williams, J., et al.; ANZGene Consortium; International Endogene Consortium; and Genetic and Environmental Risk for Alzheimer's disease Consortium (2013). Estimation and partitioning of polygenic variation captured by common SNPs for Alzheimer's disease, multiple sclerosis and endometriosis. Hum. Mol. Genet. *22*, 832–841. https://doi.org/10.1093/hmg/dds491.
6. Pang, S., Yengo, L., Nelson, C.P., Bourier, F., Zeng, L., Li, L., Kessler, T., Erdmann, J., Mägi, R., Läll, K., et al. (2023). Genetic and modifiable risk factors combine multiplicatively in common disease. Clin. Res. Cardiol. *112*, 247–257. https://doi.org/10.1007/s00392-022-02081-4.
7. Seral-Cortes, M., Sabroso-Lasa, S., De Miguel-Etayo, P., Gonzalez-Gross, M., Gesteiro, E., Molina-Hidalgo, C., De Henauw, S., Erhardt, É., Censi, L., Manios, Y., et al. (2020). Interaction Effect of the Mediterranean Diet and an Obesity Genetic Risk Score on Adiposity and Metabolic Syndrome in Adolescents: The HELENA Study. Nutrients *12*, 3841. https://doi.org/10.3390/nu12123841.
8. Carter, A.R., Harrison, S., Gill, D., Davey Smith, G., Taylor, A. E., Howe, L.D., and Davies, N.M. (2022). Educational attainment as a modifier for the effect of polygenic scores for cardiovascular risk factors: cross-sectional and prospective analysis of UK Biobank. Int. J. Epidemiol. *51*, 885–897. https://doi.org/10.1093/ije/dyac002.
9. Ye, Y., Chen, X., Han, J., Jiang, W., Natarajan, P., and Zhao, H. (2021). Interactions Between Enhanced Polygenic Risk Scores and Lifestyle for Cardiovascular Disease, Diabetes, and Lipid Levels. Circ. Genom. Precis. Med. *14*, e003128. https://doi.org/10.1161/CIRCGEN.120.003128.
10. Fu, Z., Liu, Q., Liang, J., Weng, Z., Li, W., Xu, J., Zhang, X., Xu, C., Huang, T., and Gu, A. (2022). Air pollution, genetic factors and the risk of depression. Sci. Total Environ. *850*, 158001. https://doi.org/10.1016/j.scitotenv.2022.158001.
11. Xia, X., Liu, F., Huang, K., Chen, S., Li, J., Cao, J., Yang, X., Liu, X., Shen, C., Yu, L., et al. (2023). Egg consumption and risk of coronary artery disease, potential amplification by high genetic susceptibility: a prospective cohort study. Am. J. Clin. Nutr. *118*, 773–781. https://doi.org/10.1016/j.ajcnut.2023.06.009.
12. Milani, L., Alver, M., Laur, S., Reisberg, S., Haller, T., Aasmets, O., Abner, E., Alavere, H., Allik, A., Annilo, T., et al. (2024). From Biobanking to Personalized Medicine: the journey of the Estonian Biobank. Preprint at medRxiv. https://doi.org/10.1101/2024.09.22.24313964.
13. Sapkota, Y., Steinthorsdottir, V., Morris, A.P., Fassbender, A., Rahmioglu, N., De Vivo, I., Buring, J.E., Zhang, F., Edwards, T. L., Jones, S., et al. (2017). Meta-analysis identifies five novel loci associated with endometriosis highlighting key genes involved in hormone metabolism. Nat. Commun. *8*, 15539. https://doi.org/10.1038/ncomms15539.
14. Gallagher, C.S., Mäkinen, N., Harris, H.R., Rahmioglu, N., Uimari, O., Cook, J.P., Shigesi, N., Ferreira, T., Velez-Edwards, D.R., Edwards, T.L., et al. (2019). Genome-wide association and epidemiological analyses reveal common genetic origins between uterine leiomyomata and endometriosis. Nat. Commun. *10*, 4857. https://doi.org/10.1038/s41467-019-12536-4.
15. VanderWeele, T.J., and Knol, M.J. (2014). A Tutorial on Interaction. Epidemiol. Methods *3*, 33–72. https://doi.org/10.1515/em-2013-0005.
16. Biobank UK (2014). Integrating electronic health records into the UK Biobank Resource. https://biobank.ctsy.ox.ac.uk/crystal/docs/DataLinkageProcess.pdf.
17. Pefoyo, A.J.K., Bronskill, S.E., Gruneir, A., Calzavara, A., Thavorn, K., Petrosyan, Y., Maxwell, C.J., Bai, Y., and Wodchis, W.P. (2015). The increasing burden and complexity of multimorbidity. BMC Public Health *15*, 415. https://doi.org/10.1186/s12889-015-1733-2.
18. Smith, S.M., Soubhi, H., Fortin, M., Hudon, C., and O'Dowd, T. (2012). Managing patients with multimorbidity: systematic review of interventions in primary care and community settings. Br. Med. J. *345*, e5205. https://doi.org/10.1136/bmj.e5205.
19. Montgomery, G.W., Mortlock, S., and Giudice, L.C. (2020). Should Genetics Now Be Considered the Pre-eminent Etiologic Factor in Endometriosis? J. Minim. Invasive Gynecol. *27*, 280–286. https://doi.org/10.1016/j.jmig.2019.10.020.
20. Mortlock, S., Corona, R.I., Kho, P.F., Pharoah, P., Seo, J.H., Freedman, M.L., Gayther, S.A., Siedhoff, M.T., Rogers, P.A. W., Leuchter, R., et al. (2022). A multi-level investigation of the genetic relationship between endometriosis and ovarian cancer histotypes. Cell Rep. Med. *3*, 100542. https://doi.org/10.1016/j.xcrm.2022.100542.
21. Yang, F., Wu, Y., Hockey, R., International Endometriosis Genetics Consortium, Doust, J., Mishra, G.D., Montgomery, G. W., and Mortlock, S. (2023). Evidence of shared genetic factors in the etiology of gastrointestinal disorders and endometriosis and clinical implications for disease management. Cell Rep. Med. *4*, 101250. https://doi.org/10.1016/j.xcrm.2023.101250.
22. Adewuyi, E.O., Mehta, D., Sapkota, Y., Auta, A., Yoshihara, K., Nyegaard, M., Griffiths, L.R., Montgomery, G.W., et al.; International Endogene Consortium; and 23andMe Research Team (2021). Genetic analysis of endometriosis and depression identifies shared loci and implicates causal links with gastric mucosa abnormality. Hum. Genet. *140*, 529–552. https://doi.org/10.1007/s00439-020-02223-6.
23. Zhao, B., Wang, Z., Liu, D., and Zhang, S. (2023). Genetically predicted serum testosterone and risk of gynecological disorders: a Mendelian randomization study. Front. Endocrinol. *14*, 1161356. https://doi.org/10.3389/fendo.2023.1161356.
24. Davogustto, G., Zhao, S., Li, Y., Farber-Eger, E., Lowery, B. D., Shaffer, L.L., Mosley, J.D., Shoemaker, M.B., Xu, Y., Roden, D.M., and Wells, Q.S. (2024). Unbiased characterization of atrial fibrillation phenotypic architecture provides insight to genetic liability and clinically relevant outcomes. Preprint at medRxiv. https://doi.org/10.1101/2024.02.13.24302788.
25. Sapkota, Y., Attia, J., Gordon, S.D., Henders, A.K., Holliday, E. G., Rahmioglu, N., MacGregor, S., Martin, N.G., McEvoy, M., Morris, A.P., et al. (2015). Genetic burden associated with varying degrees of disease severity in endometriosis. Mol. Hum. Reprod. *21*, 594–602. https://doi.org/10.1093/molehr/gav021.
26. Suda, K., Cruz Diaz, L.A., Yoshihara, K., Nakaoka, H., Yachida, N., Motoyama, T., Inoue, I., and Enomoto, T. (2020).

Clonal lineage from normal endometrium to ovarian clear cell carcinoma through ovarian endometriosis. Cancer Sci. *111*, 3000–3009. https://doi.org/10.1111/cas.14507.

27. Suda, K., Nakaoka, H., Yoshihara, K., Ishiguro, T., Tamura, R., Mori, Y., Yamawaki, K., Adachi, S., Takahashi, T., Kase, H., et al. (2018). Clonal Expansion and Diversification of Cancer-Associated Mutations in Endometriosis and Normal Endometrium. Cell Rep. *24*, 1777–1789. https://doi.org/10.1016/j.celrep.2018.07.037.
28. Anglesio, M.S., Papadopoulos, N., Ayhan, A., Nazeran, T.M., Noë, M., Horlings, H.M., Lum, A., Jones, S., Senz, J., Seckin, T., et al. (2017). Cancer-Associated Mutations in Endometriosis without Cancer. N. Engl. J. Med. *376*, 1835–1848. https://doi.org/10.1056/NEJMoa1614814.
29. Zhang, L., and Yan, H. (2024). Genetically identification of endometriosis and cancers risk in women through a two-sample Mendelian randomization study. Sci. Rep. *14*, 8382. https://doi.org/10.1038/s41598-024-58950-7.
30. Uimari, O., Nazri, H., and Tapmeier, T. (2021). Endometriosis and Uterine Fibroids (Leiomyomata): Comorbidity, Risks and Implications. Front. Reprod. Health *3*, 750018. https://doi.org/10.3389/frph.2021.750018.

HGGA, Volume *6*

Supplemental information

Interaction between genetic risk

and comorbid conditions in endometriosis

Isabelle M. McGrath, Valentina Rukins, Triin Laisk, Estonian Biobank Research Team, Sally Mortlock, and Grant W. Montgomery

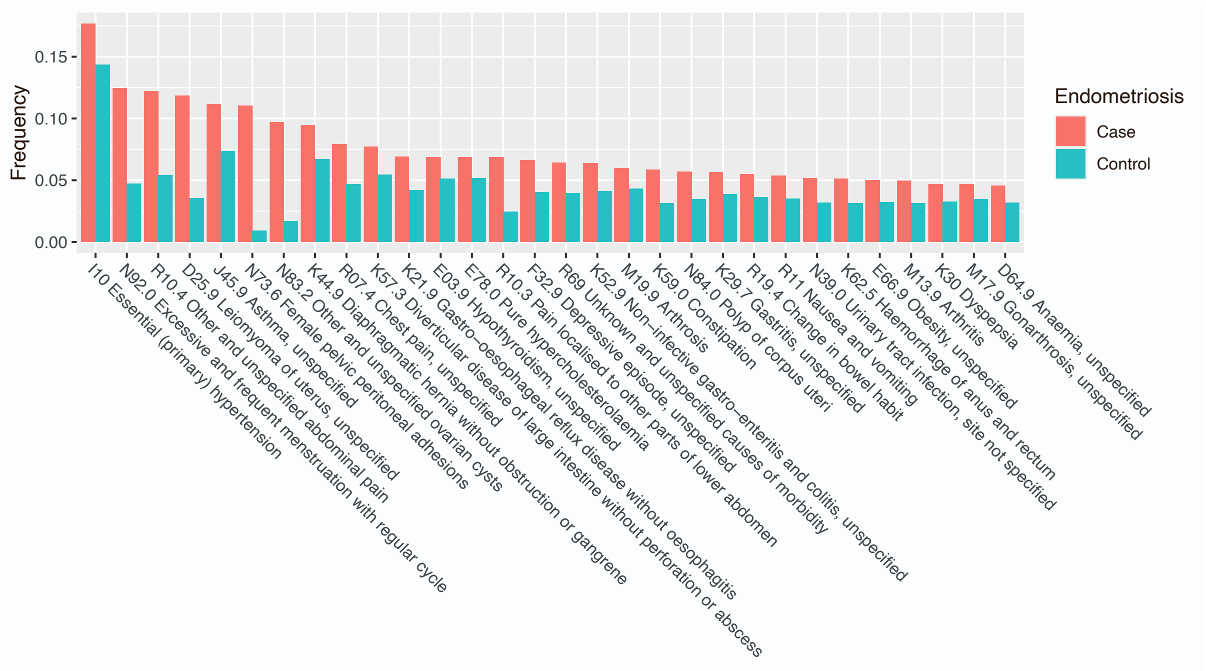

Figure S1. Prevalence of ICD10 codes significantly associated with endometriosis in 5,432 unrelated European ancestry endometriosis cases and 92,344 age matched female controls in the UK Biobank. The 30 most frequently occurring codes in endometriosis cases are shown, ordered by frequency in cases. ICD10 codes related to external factors have been excluded.

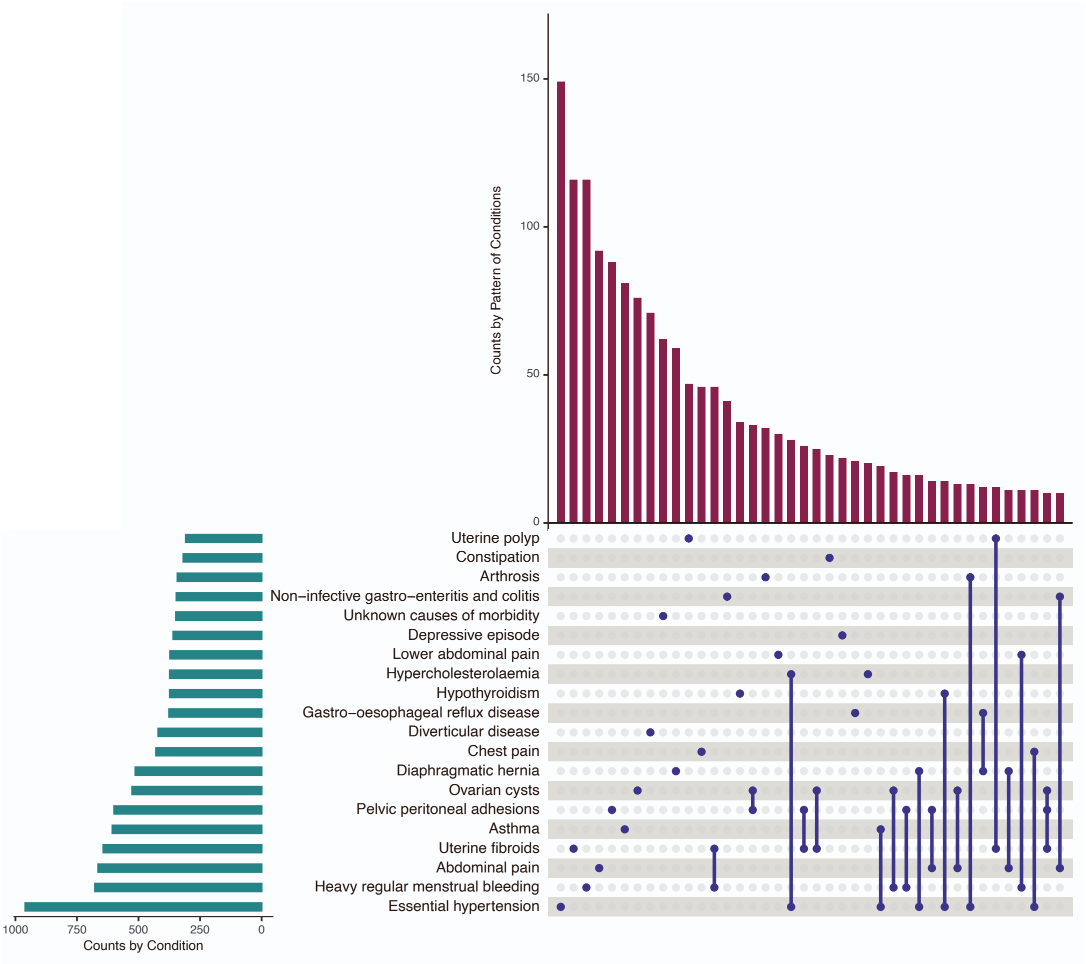

Figure S2. UpSet plot of the most common trait combinations associated with endometriosis in 5,432 endometriosis cases in the UK Biobank. Essential hypertension: I10 Essential (primary) hypertension; Heavy regular menstrual bleeding: N92.0 Excessive and frequent menstruation with regular cycle; Abdominal pain: R10.4 Other and unspecified abdominal pain; Uterine fibroids: D25.9 Leiomyoma of uterus, unspecified; Asthma: J45.9 Asthma, unspecified; Pelvic peritoneal adhesions: N73.6 Female pelvic peritoneal adhesions; Ovarian cysts: N83.2 Other and unspecified ovarian cysts; Diaphragmatic hernia: K44.9 Diaphragmatic hernia without obstruction or gangrene; Chest pain: R07.4 Chest pain, unspecified; Diverticular disease: K57.3 Diverticular disease of large intestine without perforation or abscess; Gastro-oesophageal reflux disease: K21.9 Gastro−oesophageal reflux disease without oesophagitis; Hypothyroidism: E03.9 Hypothyroidism, unspecified; Hypercholesterolaemia: E78.0 Pure hypercholesterolaemia; Lower abdominal pain: R10.3 Pain localised to other parts of lower abdomen; Depressive episode: F32.9 Depressive episode, unspecified; Unknown causes of morbidity: R69 Unknown and unspecified causes of morbidity; Non−infective gastro−enteritis and colitis : K52.9 Non−infective gastro−enteritis and colitis, unspecified; Arthrosis: M19.9 Arthrosis; Constipation: K59.0 Constipation; Uterine polyp: N84.0 Polyp of corpus uteri.

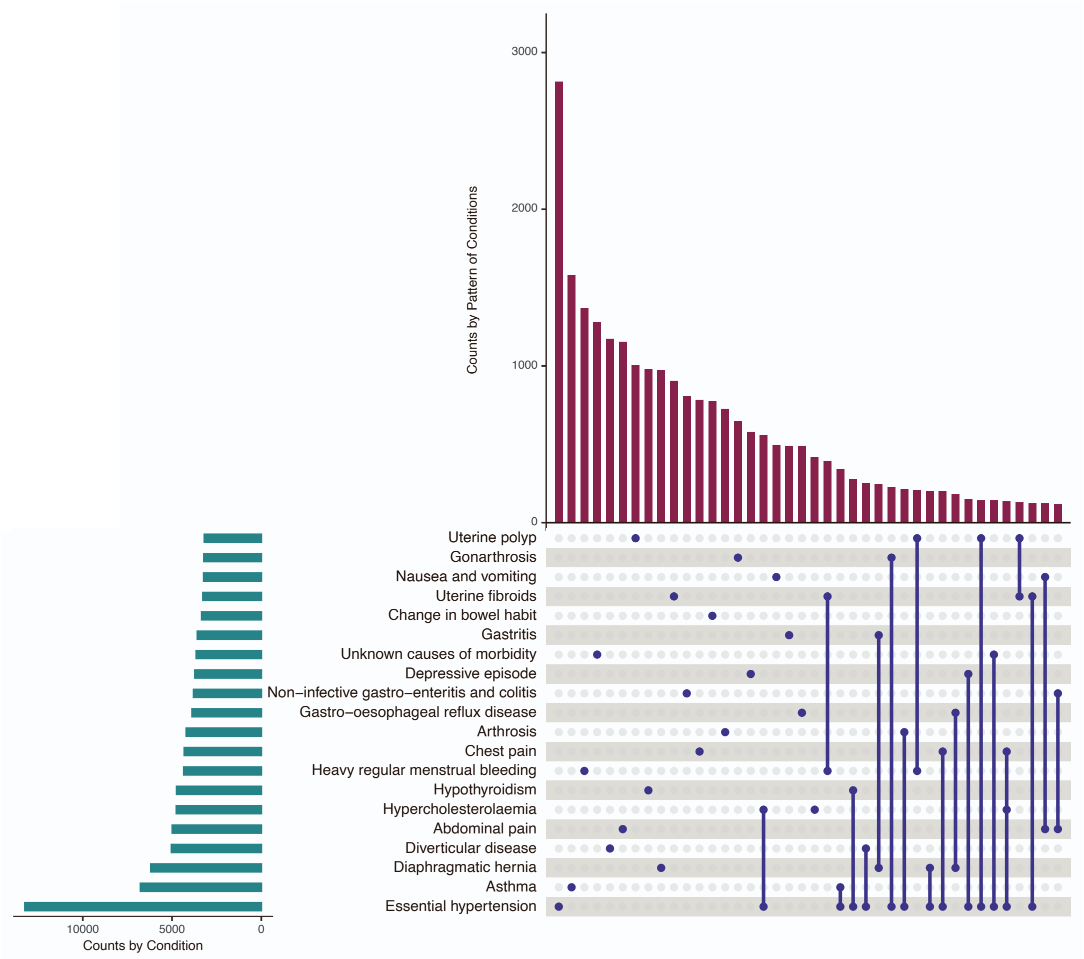

Figure S3. UpSet plot of most common trait combinations associated with endometriosis in 92,344 endometriosis controls in the UK Biobank. Essential hypertension: I10 Essential (primary) hypertension; Asthma: J45.9 Asthma, unspecified; Diaphragmatic hernia: K44.9 Diaphragmatic hernia without obstruction or gangrene; Diverticular disease: K57.3 Diverticular disease of large intestine without perforation or abscess; Abdominal pain: R10.4 Other and unspecified abdominal pain; Hypercholesterolaemia: E78.0 Pure hypercholesterolaemia; Hypothyroidism: E03.9 Hypothyroidism, unspecified; Heavy regular menstrual bleeding: N92.0 Excessive and frequent menstruation with regular cycle; Arthrosis: M19.9 Arthrosis; Chest pain: R07.4 Chest pain, unspecified; Gastro−oesophageal reflux disease: K21.9 Gastro−oesophageal reflux disease without oesophagitis; Non−infective gastro−enteritis and colitis: K52.9 Non−infective gastro−enteritis and colitis, unspecified; Depressive episode: F32.9 Depressive episode, unspecified; Unknown causes of morbidity: R69 Unknown and unspecified causes of morbidity; Gastritis: K29.7 Gastritis, unspecified; Change in bowel habit: R19.4 Change in bowel habit; Uterine fibroids: D25.9 Leiomyoma of uterus, unspecified; Nausea and vomiting: R11 Nausea and vomiting; Gonarthrosis: M17.9 Gonarthrosis, unspecified; Uterine polyp: N84.0 Polyp of corpus uteri.

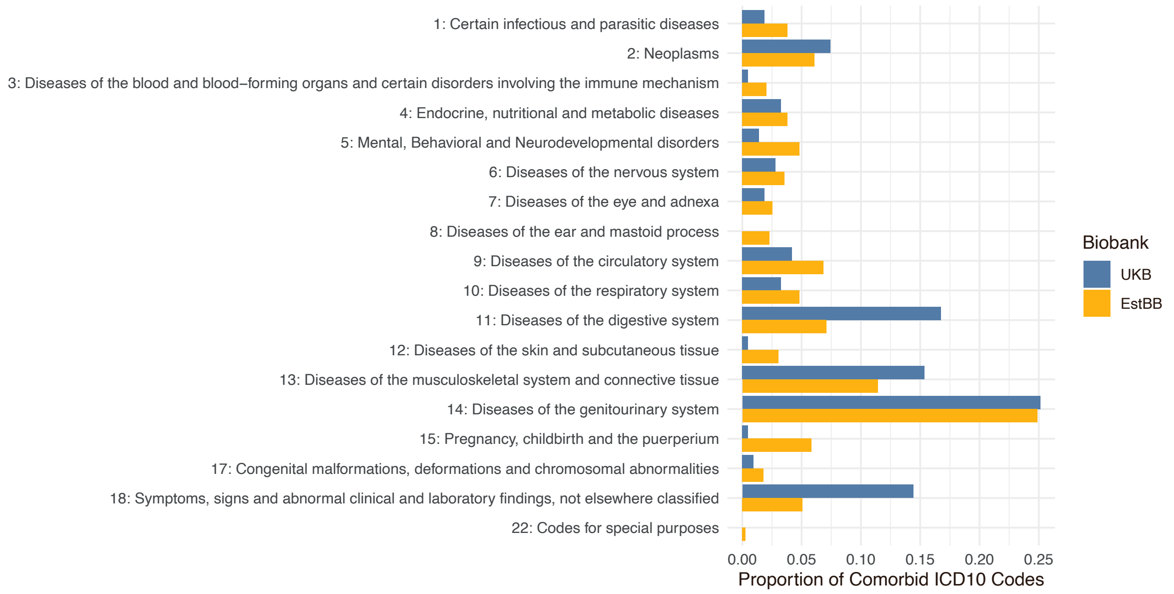

Figure S4. Chapter distribution of ICD10 codes identified as comorbid with endometriosis by biobank. EstBB = Estonian Biobank, UKB = UK Biobank.

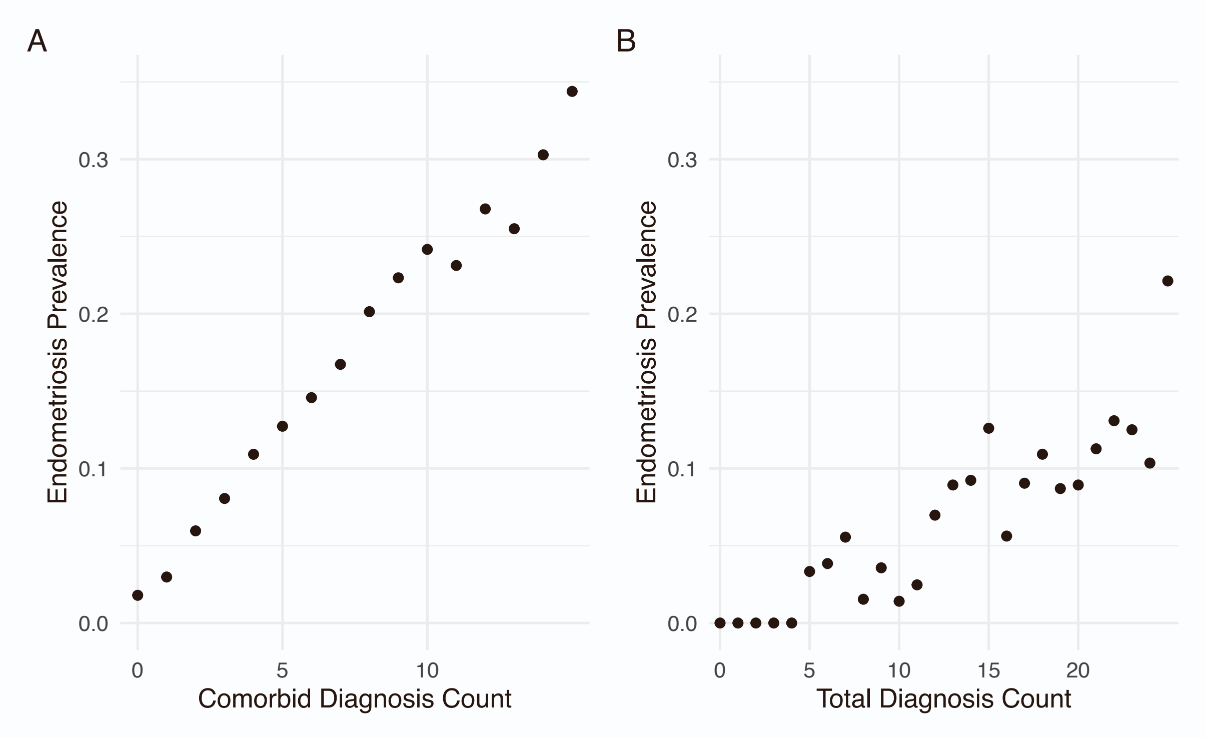

Figure S5. Endometriosis prevalence by comorbidity burden in 3,824 unrelated European ancestry endometriosis cases and 15,296 age matched female controls from the Estonian Biobank. A. Known endometriosis comorbidities (identified in UKB) only. B. All diagnosed conditions.

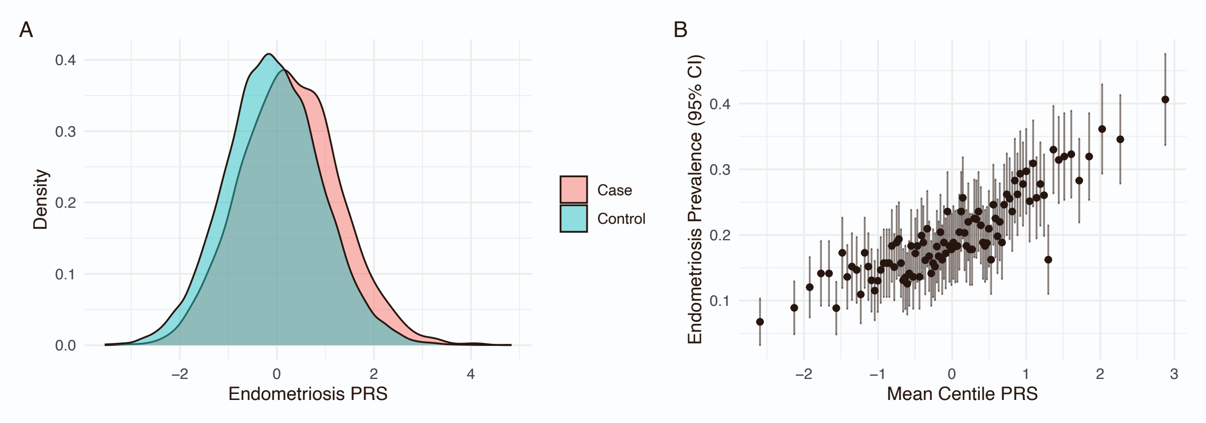

Figure S6. Density plot of endometriosis polygenic risk scores in 3,824 endometriosis cases and 15,296 female controls (unrelated European) in the Estonian Biobank. B. Odds ratio (95% CI) of endometriosis by endometriosis PRS centile compared to individuals in the 50th centile. The mean PRS of each centile is plotted on the x axis.
